# The influence of pyroptosis-related genes on the development of chronic obstructive pulmonary disease

**DOI:** 10.1186/s12890-023-02408-5

**Published:** 2023-05-17

**Authors:** Xinlong Liu, Xiaoling Huang, Feng Xu

**Affiliations:** Department of Intensive Care Unit, University of Chinese Academy of Sciences-Shenzhen Hospital, Shenzhen, Guangdong China

**Keywords:** Chronic obstructive pulmonary disease, Pyroptosis-related genes, Occurrence, Development, Immune environment

## Abstract

Increasing evidences have demonstrated that pyroptosis exerts key roles in the occurrence, development of chronic obstructive pulmonary disease. However, the mechanisms of pyroptosis in COPD remain largely unknown. In our research, Statistics were performed using R software and related packages in this study. Series matrix files of small airway epithelium samples were downloaded from the GEO database. Differential expression analysis with FDR < 0.05 was performed to identify COPD-associated pyroptosis-related genes. 8 up-regulated genes (CASP4, CASP5, CHMP7, GZMB, IL1B, AIM2, CASP6, GSDMC) and 1 down-regulated genes (PLCG1) was identified as COPD-associated pyroptosis-related genes. Twenty-six COPD key genes was identified by WGCNA analysis. PPI analysis and gene correlation analysis showed their relationship clearly. KEGG and GO analysis have revealed the main pyroptosis-related mechanism of COPD. The expression of 9 COPD-associated pyroptosis-related genes in different grades was also depicted. The immune environment of COPD was also explored. Furthermore, the relationship of pyroptosis-related genes and the expression of immune cells was also be shown in the end. In the end, we concluded that pyroptosis influences the development of COPD. This study may provide new insight into the novel therapeutic targets for COPD clinical treatment.

**Supplementary Information:**

The online version contains supplementary material available at 10.1186/s12890-023-02408-5.

## Introduction

Chronic obstructive pulmonary disease (COPD) is a common chronic respiratory disease characterized by persistent respiratory symptoms, progressive airflow limitation. Because of the lack of effective treatment, this disease leads to high mortality [1]. Despite significant progress in the treatment and prevention of acute exacerbations, the advances in ameliorating disease progression and mortality are limit. Thus, a greater understanding of the mechanisms resulting in COPD is urgently needed [2]. At present, some evidences reported that pyroptosis has been considered to be involved in the occurrence and development of COPD [3].Inhibition of pyroptosis in respiratory system could reduce the injury of lung cells, the production of inflammatory factors and inflammatory response [4].

Pyroptosis, an inflammatory programmed cell death pathway, is considered as a non-specific defense mechanism of the body [5]. The activation of pyroptosis mainly involves two pathways [6].It is currently recognized that the classical pathway is the activation of the inflammasome Caspase-1, which promotes the cleavage of gasdermin D (GSDMD) and the release of IL-1β and IL-18 [7, 8]. The noncanonical pathway is the activation of the caspase-4/5 regulated by cytosolic bacterial lipopolysaccharide (LPS) [9].Currently, known inflammasomes include the NLRP1, NLRP3, NLRC4, AIM2, and the PYRIN inflammasome., which have been studied intensively. Following prior researches, numerous factors activate the inflammasomes, including exogenous and endogenous danger signals [7]. Inflammasomes are involved in host immune defense, but excessive inflammatory damage and pyroptosis will leads to irreversible injuries. As reported, inflammasomes are involed in both COPD stable and exacerbations [10].Inhalation of harmful particles and gases activate the pattern recognition receptors in the lungs,then trigger downstream signaling pathways of the nuclear factor (NF)-κB, which lead to airway inflammation in COPD [11]. Caspase-1, a cysteine protease, is located downstream of inflammatory pathway to activate Gasdermin-D [12]. The N-terminal domains of cleaved Gasdermin-D lead to a loss of membrane integrity, which induce pyroptosis eventually [13]. Furthermore, activated inflammasomes like NLRP3、NLRC4 and AIM2 also influence trigger the activation of caspase-1, which cleaves the cytokine precursors interleukin-1B (pro-IL-1B) and interleukin-18 (pro-IL-18) to active pro-inflammatory factors IL-1B and IL-18 [14].

The aim of this study was to identify key genes in COPD and to investigate the relationship between COPD key genes and pyroptosis-related genes by integrating bioinformatics tools. Also, the correlation between pyroptosis-related genes and immune infiltration have been explored. This study also provides new insight into the novel therapeutic targets for COPD clinical treatment.

## Materials and methods

### Information of COPD patients

The datasets of our research were downloaded from the Gene Expression Omnibus (GEO) database (https://www.ncbi.nlm.nih.gov/geo/). The retrieval condition is set with the subject word of "chronic obstructive pulmonary disease", the research type of "expression profiling by array", and the species of "homo sapiens". Series matrix files of small airway epithelium samples obtained by fiberoptic bronchoscopy including GSE5058, GSE8545, GSE11906, GSE20257 and GSE69818 were selected. To eliminate batch effects, R package “sva” and “limma” were applied for batch effect normalization [15]. Therefore, the train set consisted of GSE8545 and GSE20257, which contained 95 healthy controls and 27 COPD patients. The test set was comprised by GSE5058 and GSE11906 including 81 healthy controls and 45 COPD cases. GSE69818 consisted of 70 former smokers with COPD including 9 cases with The Global Average of COPD (GOLD) stage 1, 41 cases with GOLD stage 2, 9 cases with GOLD stage 3, and 9 cases with GOLD stage 4.

### PPI analysis and gene correlation alalysis

We performed protein–protein interaction (PPI) analysis by the STRING database (http://www.strin g-db.org/). The R package “limma” and “corrplot” was applied to perform differential expression analysis. The expression of 9 COPD-associated pyroptosis-related genes in different GOLD states was revealed using GSE69818, which completed by R packages “limma”,”ggplot2″ and “ggpubr” [16].

### Identification of COPD-associated pyroptosis-related genes

According to the previous researches for pyroptosis [17–21], the expression matrixes of 47 pyroptosis-related genes were extracted. Differential expression analysis for 47 pyroptosis-related genes between COPD patients and health cases was conducted by wilcoxtest [22].

### Identification of COPD key genes

Using the “limma” package, the differentially expressed genes (DEGs) between COPD patients and health controls for the train set and test set were respectively identified. DEGs were filtered by |log2 fold change (FC)|> 1 and adjust *P* value < 0.05. The overlapped DEGs were acquired [23].

The DEGs (FDR < 0.05) obtained from test set were uesd to identify the hub COPD genes by weighted correlation network analysis (WGCNA) on mRNA expression data of train set [23]. The adjacency matrix was transformed into the topological overlap matrix (TOM) when the power was equal to 8 (${R}^{2}$= 0.9) [24]. Filtered by the cor. COPD > 0.2 and the cor. module membership > 0.6, the hub genes were obtained. The intersectant genes of the overlapped DEGs and hub COPD genes were defined as key COPD genes.

### KEGG and GO enrichment analyses

Kyoto Encyclopedia of Genes and Genomes (KEGG) pathway enrichment and Gene Ontology (GO) functional analyses were conducted to analyze 9 COPD-associated pyroptosis-related genes and 26 COPD key genes by R packages “colorspace”, "stringi" and “ggplot2”. The *P* value < 0 0.05 and q value < 0 0.05 were set as the cutoff criterion [25–27].

### Evaluation of tissue-infiltrating immune cells

The R package “CIBERSORT.R” was used to estimate the relative proportions of 22 types of immune cells [28]. CIBERSORT, a kind of deconvolution algorithm, transform the normalized gene expression matrix into the corresponding infiltrating immune cell expression matrix, which provide a reliable composition of infiltrating immune cells. The results were visualized using the R packages “corrplot”, “vioplot”, “ggplot2”, and “dplyr” [29].

## Result

### Expression of pyroptosis-related genes in COPD

**Fig. 1:**
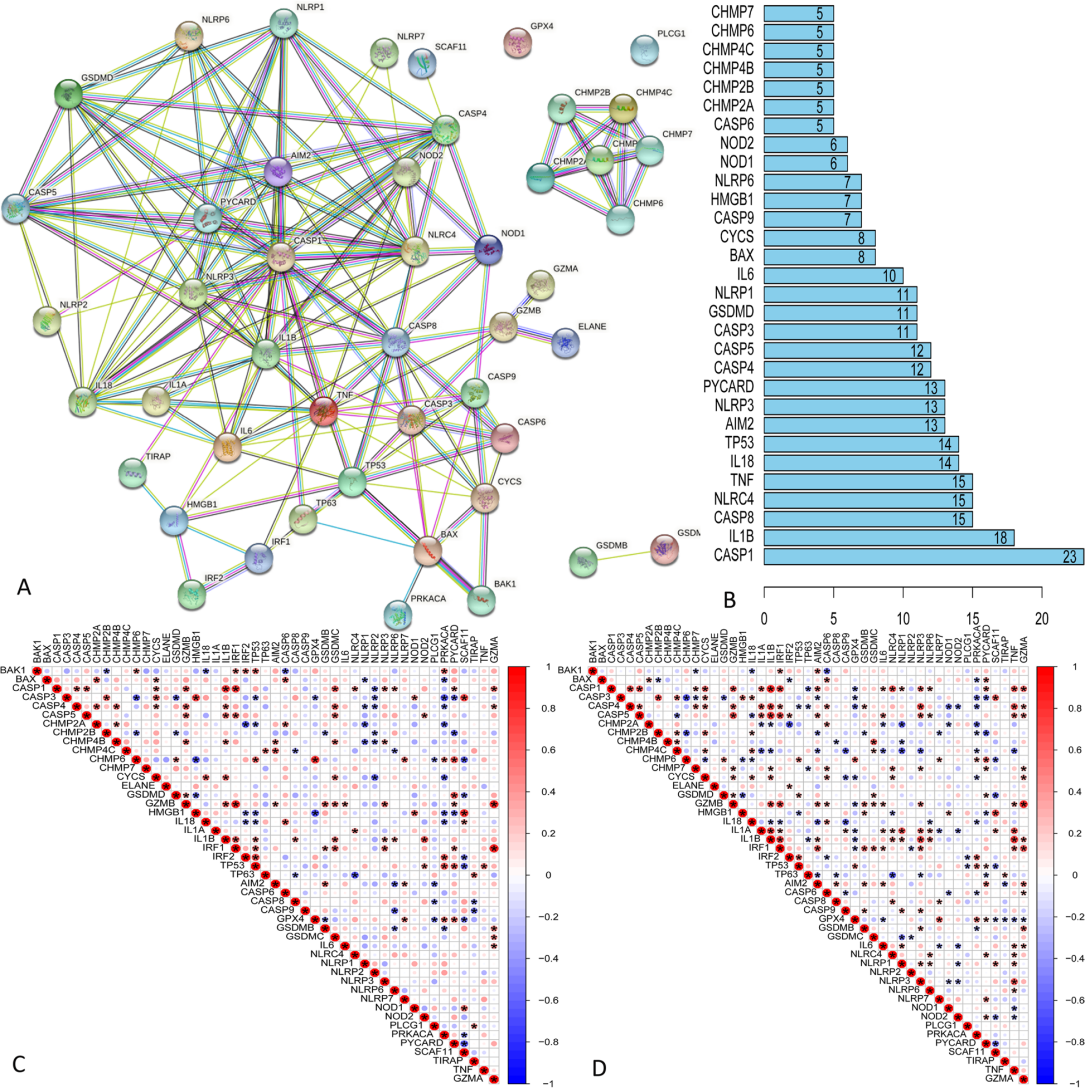
PPI analysis and gene correlation analysis for 47 pyroptosis-related genes. **A** The protein–protein interactions among 47 pyroptosis-related genes. **B** The rank of connection degree (number) for each genes. **C** Correlation among the expression of 47 pyroptosis-related gene in the train set. P values are shown as: *p < 0.05. **D** Correlation among the expression of 47 pyroptosis-related genes in the test set. P values are shown as: *p < 0.05

Forty-seven pyroptosis-related genes have showed in Supplemental Table 1. We performed PPI analysis to further understand biological interactions among 47 pyroptosis-related genes (Fig. 1A). Figure 1B showed that these pyroptosis-related genes presented highly significant correlations. Furthermore, we investigated the expression correlation among these pyroptosis-related genes. The result we obtained showed that a basically consistent correlation among 47 pyroptosis-related gene in both train set and test set (Fig. 1C, D).

**Fig. 2:**
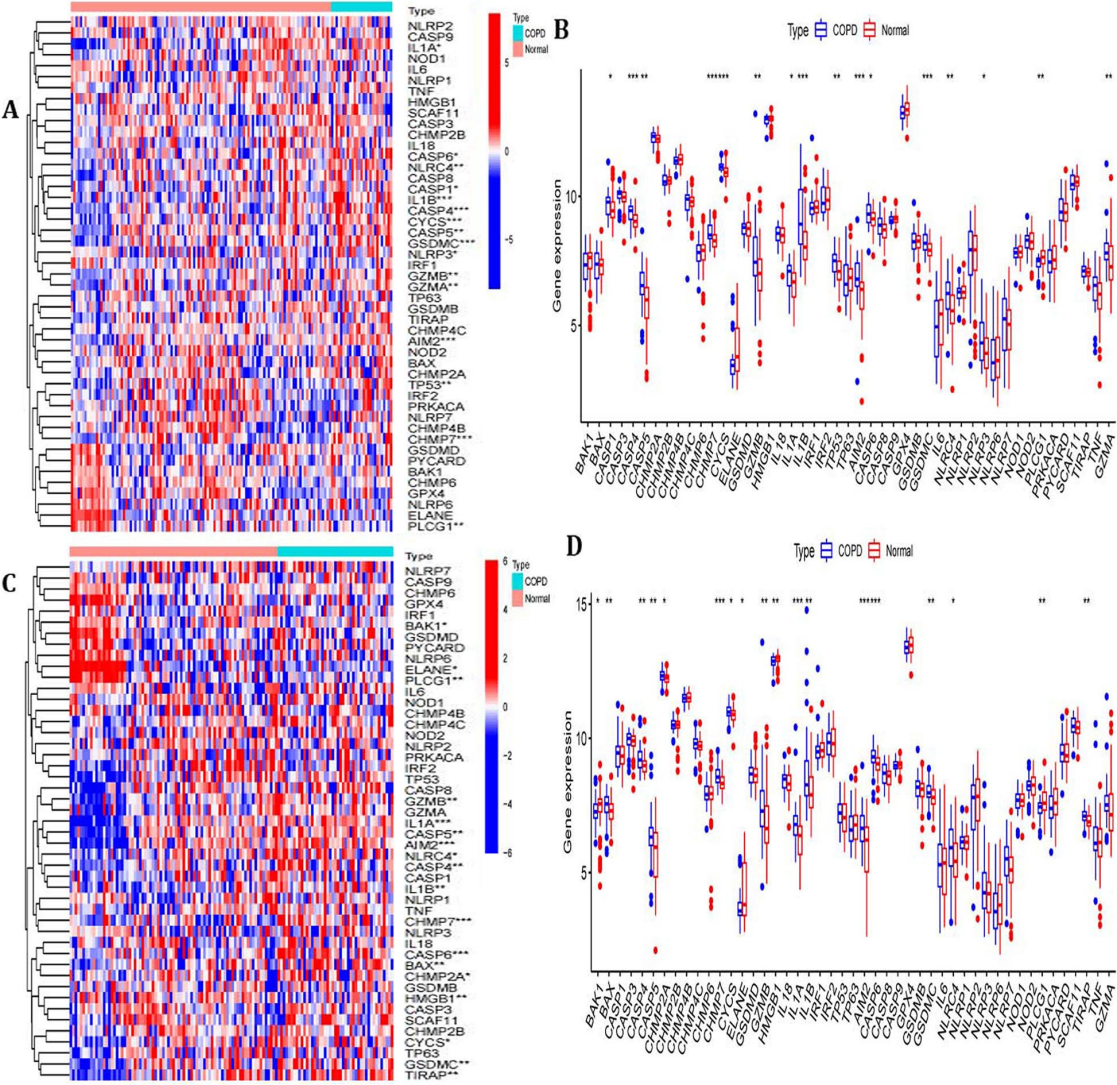
Expression of pyroptosis-related genes in COPD. **A**-**B** Heatmap and boxplot of expression levels of 47 pyroptosis-related genes in train set. *P*-values are shown as: *p < 0.05; **p < 0.01; ***p < 0.001; (**C**-**D**) Heatmap and boxplot of expression levels of 47 pyroptosis-related genes from test set. *P*-values are shown as: *p < 0.05; **p < 0.01; ***p < 0.001

The clinical information of samples have revealed in Supplemental Table 2. The differential expression analysis for 47 pyroptosis-related gene between the COPD patients and healthy cases was conducted in the train set (Fig. 2A-B) and test set (Fig. 2C-D). Therefore, these results indicated that the expression of pyroptosis-related gene may influence the occurrence of COPD. Based on the FDR < 0.05, we found that 9 pyroptosis-related genes were related to the occurrence of COPD significantly, with 8 up-regulated (CASP4, CASP5, CHMP7, GZMB, IL1B, AIM2, CASP6, GSDMC) and 1 down-regulated genes (PLCG1) in COPD patients in both train set and test set (Supplemental Table 3). And we then also named these 9 pyroptosis-related genes as COPD-associated pyroptosis-related genes.

### Identification of DEGs in COPD

Filtered by |logFC|> 1 and FDR < 0.05, 89 DEGs including 17 down-regulated and 72 up-regulated genes were acquired from the train set. Similarly, 86 DEGs including 36 down-regulated and 50 up-regulated genes were acquired from the test set. Finally, we got 38 overlapped DEGs.

### Identification of the key genes

**Fig. 3:**
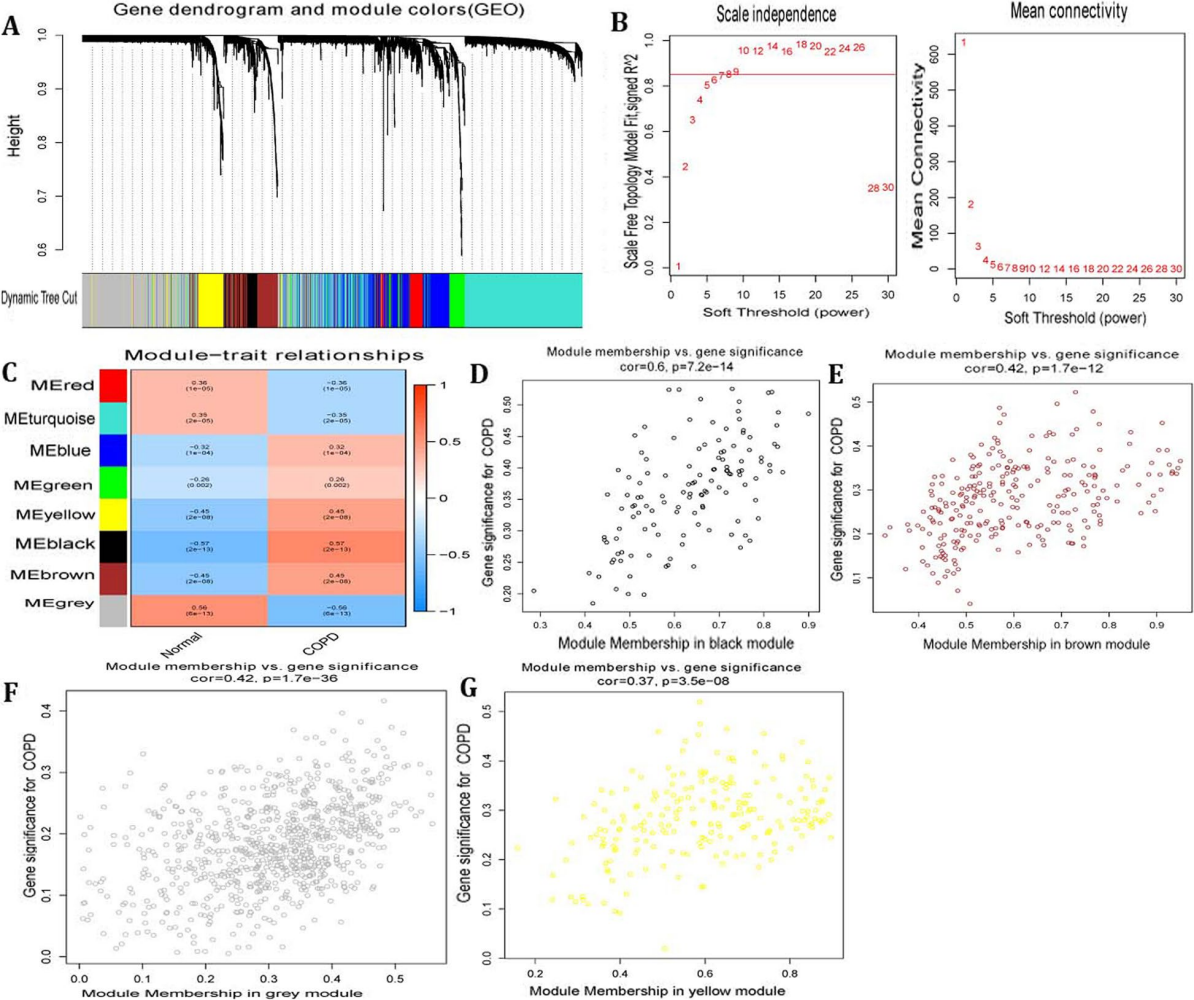
Identification of Key genes. **A** Clustering dendrograms of genes based on a dissimilarity measure (1-TOM). **B** Analysis of the scale-freefit index (left) and the mean connectivity (right) for various soft-thresholding powers. **C** Module-trait associations were evaluated by correlations between module eigengenes and sample traits. **D**-**F** Scatterplot of Gene Significance for COPD in black, brown, grey, yellow module

To identify the modules associated with the occurrence of COPD, we conducted WGCNA with the train set for 4308 genes acquired from test set filtered by FDR < 0.05. A soft-thresholding power of 8, for which the scale-free topology fit index reaches 0.90 (Fig. 3B), which was applied to establish a hierarchical clustering tree (Fig. 3A).

We analyzed the module trait relationship to further investigate the modules associated with COPD. Subsequently, eight modules were identified (Fig. 3C). The correlation between ME and traits shows that some modules are more important than others in the COPD. On the basis of cor. COPD > 0.2 and cor. module membership > 0.6, 213 hub COPD genes with the high connectivity in black, brown, green and yellow modules (Fig. 3D-G) were selected out. By taking the intersection of 38 overlapped DEGs and 213 hub COPD genes. Finally, 26 COPD key genes were acquired (Table 1).

**Table 1:**
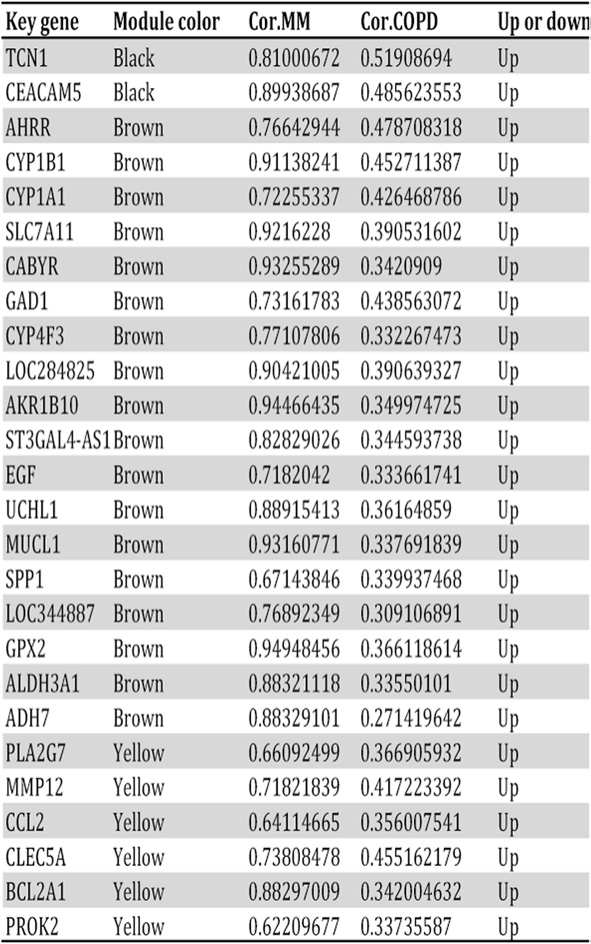
Twenty-six COPD key genes positively or negatively associated with COPD and each MM

### KEGG and GO enrichment analyses

**Fig. 4:**
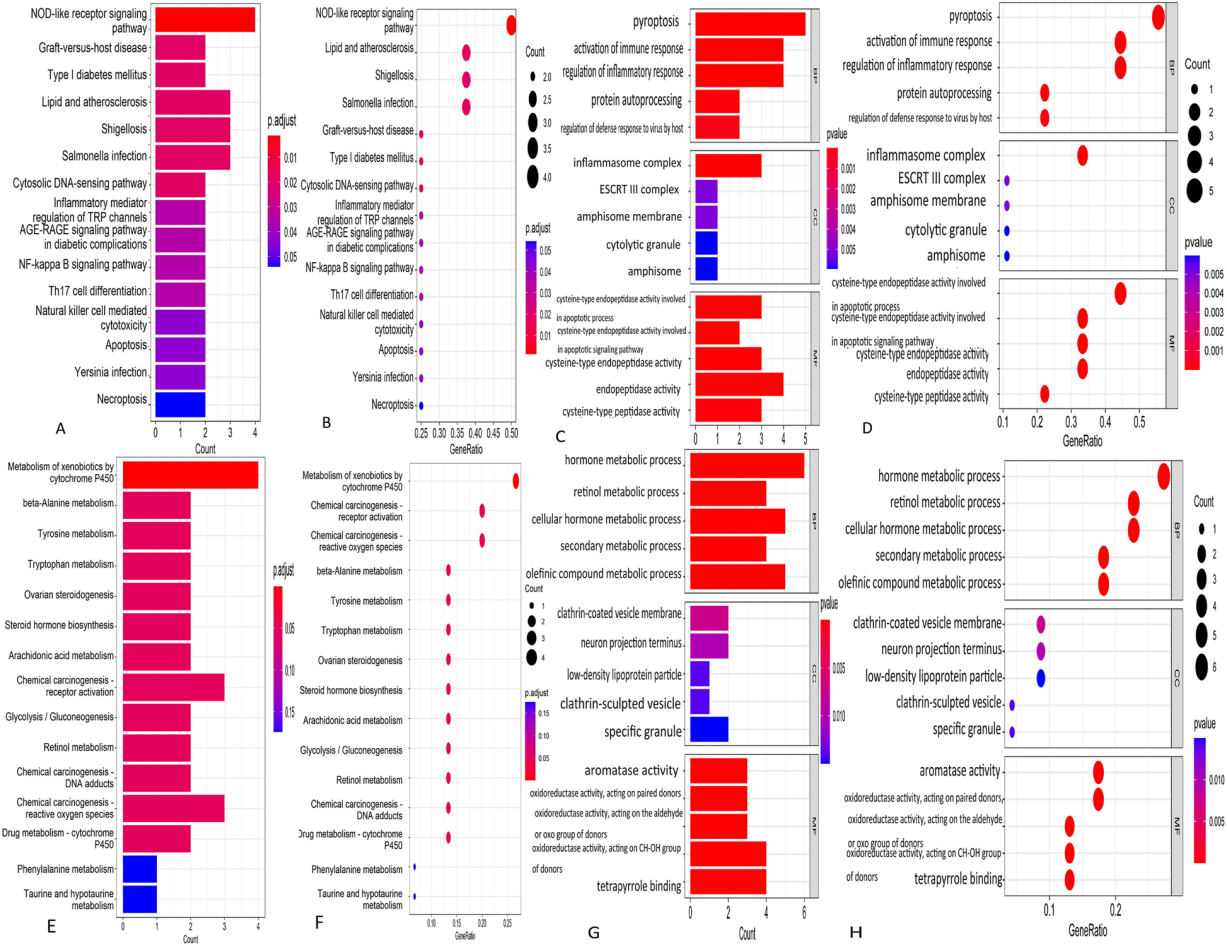
KEGG and GO enrichment analysis. **A**-**B** KEGG analysis for 9 COPD-associated pyroptosis-related genes. **C**-**D** Go analysis for 9 COPD-associated pyroptosis-related genes. (E–F) KEGG analysis for 26 COPD key genes. **G**-**H** Go analysis for 26 COPD key genes

KEGG analysis (Fig. 4A-B) for 9 COPD-associated pyroptosis-related genes indicated that these genes were significantly enriched in NOD-like receptor signaling pathway (CASP4/CASP5/IL1B/AIM2), Lipid and atherosclerosis (IL1B/PLCG1/CASP6), Shigellosis (CASP4/IL1B/PLCG1), Salmonella (CASP4/CASP5/PLCG1) infection and so on (Fig. 4A). As reported, NOD-like receptor is an important family of intracellular pattern recognition receptors and the key component of the first defense against pathogen attacks, which has played a vital role in the pathogenesis and AECOPD. The results of GO analysis (Fig. 4C-D) for 9 COPD-associated pyroptosis-related genes demonstrated that these genes were involed in pyroptosis (CASP4/GZMB/AIM2/CASP6/GSDMC), activation of immune response (IL1B/AIM2/CASP6/PLCG1), regulation of inflammatory response (regulation of inflammatory response) and endopeptidase activity (CASP4/CASP5/GZMB/CASP6). The detailed results of GO analysis for 9 COPD-associated pyroptosis-related genes have revealed in Supplemental Table 4.

Moreover, we also performed KEGG analysis (Fig. 4E-F) and Go analysis (Fig. 4G-H) for 26 COPD key genes. KEGG analysis for 26 COPD key genes indicated that metabolism of xenobiotics by cytochrome P450 (CYP1B1/CYP1A1/ALDH3A1/ADH7), Chemical carcinogenesis—DNA adductsand (CYP1B1/CYP1A1/EGF) and chemical carcinogenesis—receptor activation (CYP1B1/CYP1A1/EGF) were related to COPD. Moreover, Go analysis showed that 26 COPD key genes enriched in 299 biological processes, 17 cellular component and 55 molecular function, which indicated that these biological processes and molecular functions may contribute to the occurrence and process of COPD. The detailed results of GO analysis for 26 COPD key genes have revealed in Supplemental Table 5.

### PPI analysis and gene correlation analysis

**Fig. 5:**
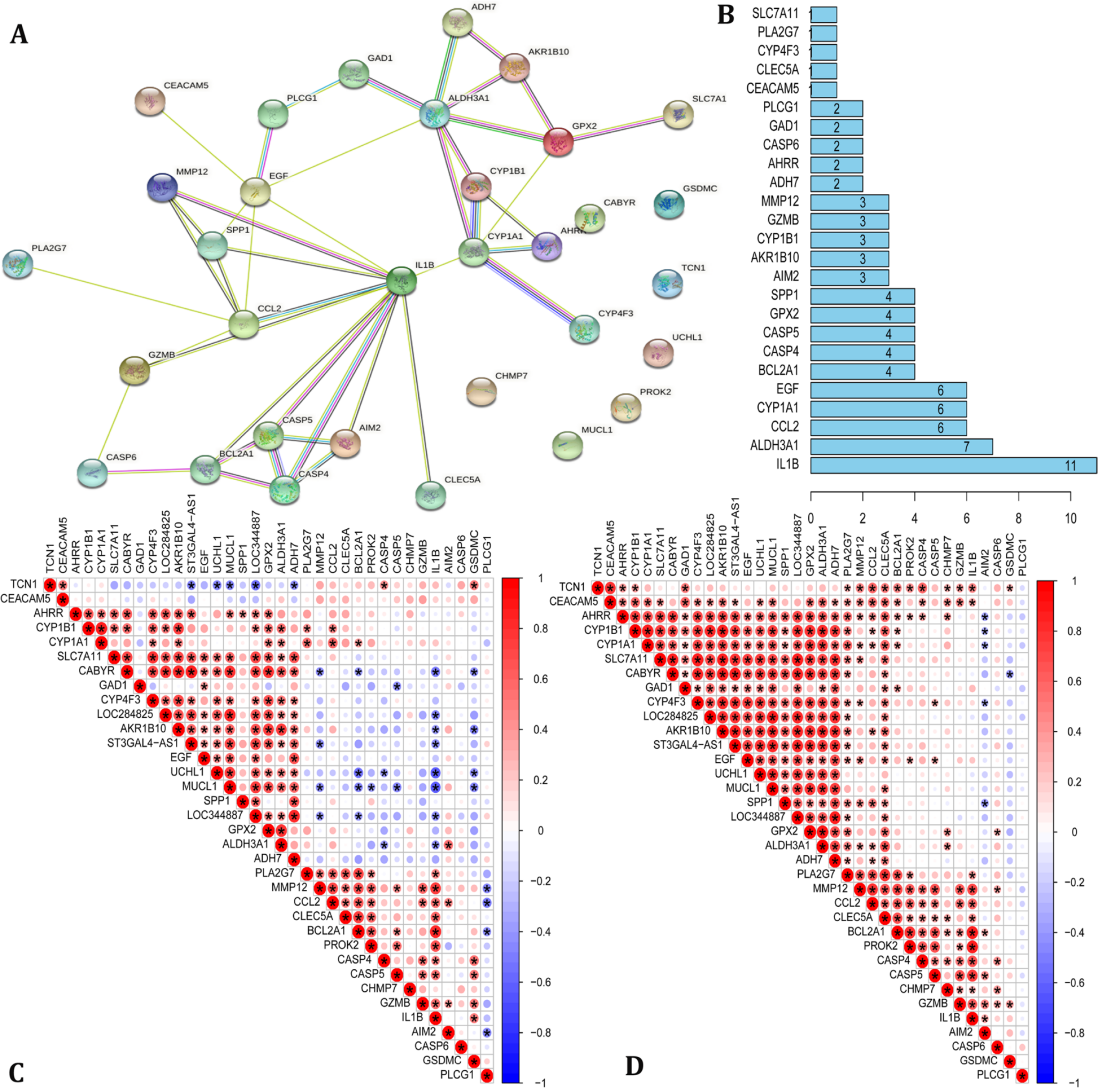
PPI analysis and gene correlation analysis. **A**The protein–protein interactions among 9 COPD-associated pyroptosis-related genes and 26 COPD key genes. **B** The rank of connection degree (number) for each gene. **C** Correlation among the expression of 9 COPD-associated pyroptosis-related genes and 26 COPD key genes in the train set. *P* values are shown as: *p < 0.05. (D) Correlation among the expression of 9 COPD-associated pyroptosis-related genes and 26 COPD key genes in the test set. P values are shown as: *p < 0.05

PPI analysis and gene correlation analysis were applied to explore the relationship between 9 COPD-associated pyroptosis-related genes and 26 COPD key genes. PPI analysis (Fig. 5A-B) show that these pyroptosis-related genes, especially IL1B and IL1A could interact with these COPD key genes. We also performed gene correlation analysis for 9 COPD-associated pyroptosis-related genes and 26 COPD key genes by the train set and test set. In the train set (Fig. 5C) and test set (Fig. 5D), the result showed that CASP5 and ILB exhibited a positive correlation with the expression of MMP12 and BCL2A1. Moreover, ILB also related to the expression of PLA2G7, CCL2, CLEC5A. CASP4 was positively associated with TCN1. GZMB have a positive correlation with PLA2G7. GSDMC related to CABYR negatively.

### Exploration the relationship between pyroptosis-related genes and GOLD states

**Fig. 6:**
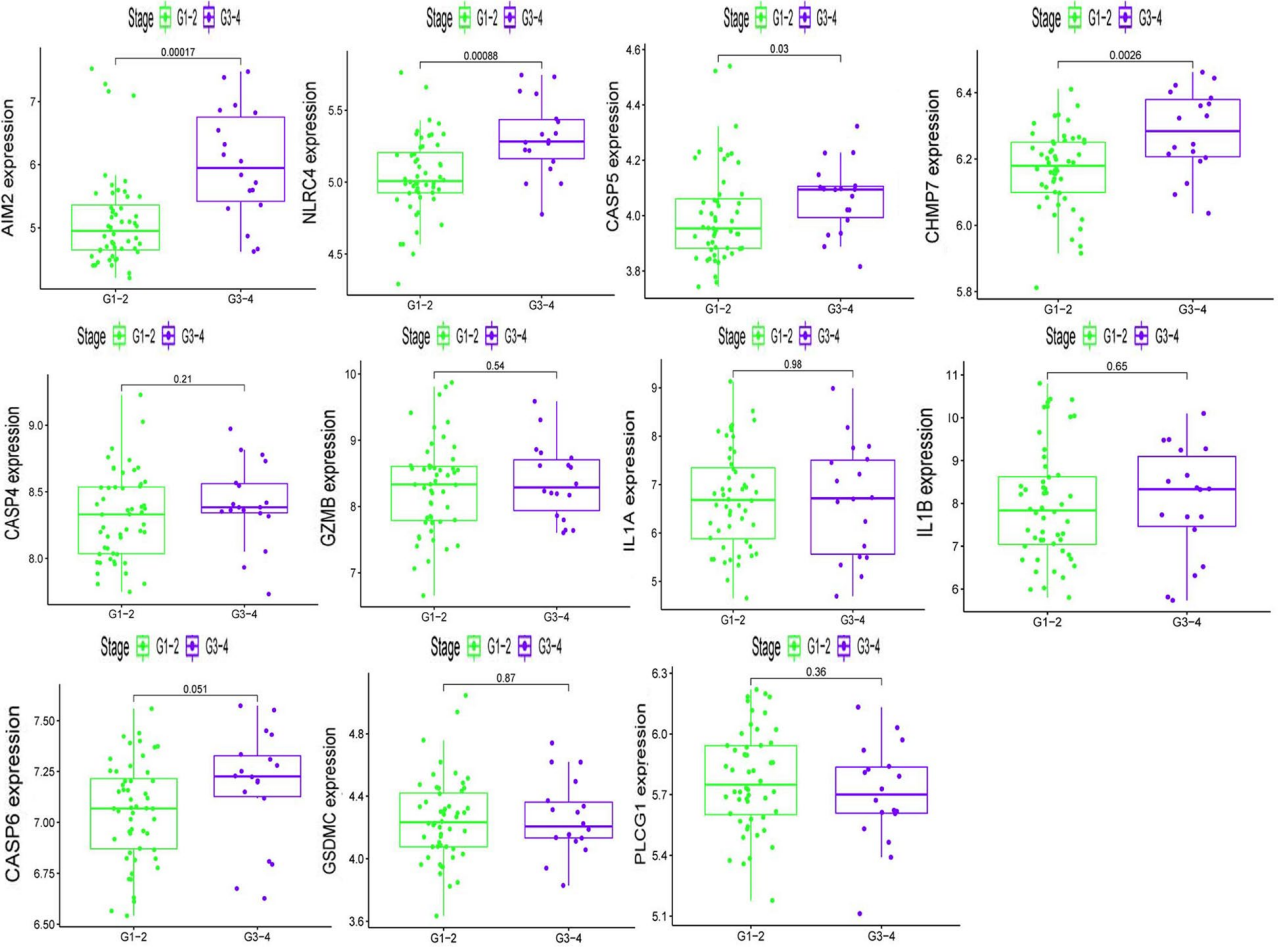
Exploration the relationship between pyroptosis-related genes and GOLD states. The expression of 9 COPD-associated pyroptosis-related genes in different GOLD states

To evaluate whether pyroptosis-related genes has an effect on COPD progression, we also explored the relationship between 9 COPD-associated pyroptosis-related genes and GOLD states (Fig. 6). As the result, the expression of AIM2, CASP5, CHMP7 have significant differences in different GOLD states, which suggested these genes may contribute to the development of COPD.

### Immune Landscape of COPD

**Fig. 7:**
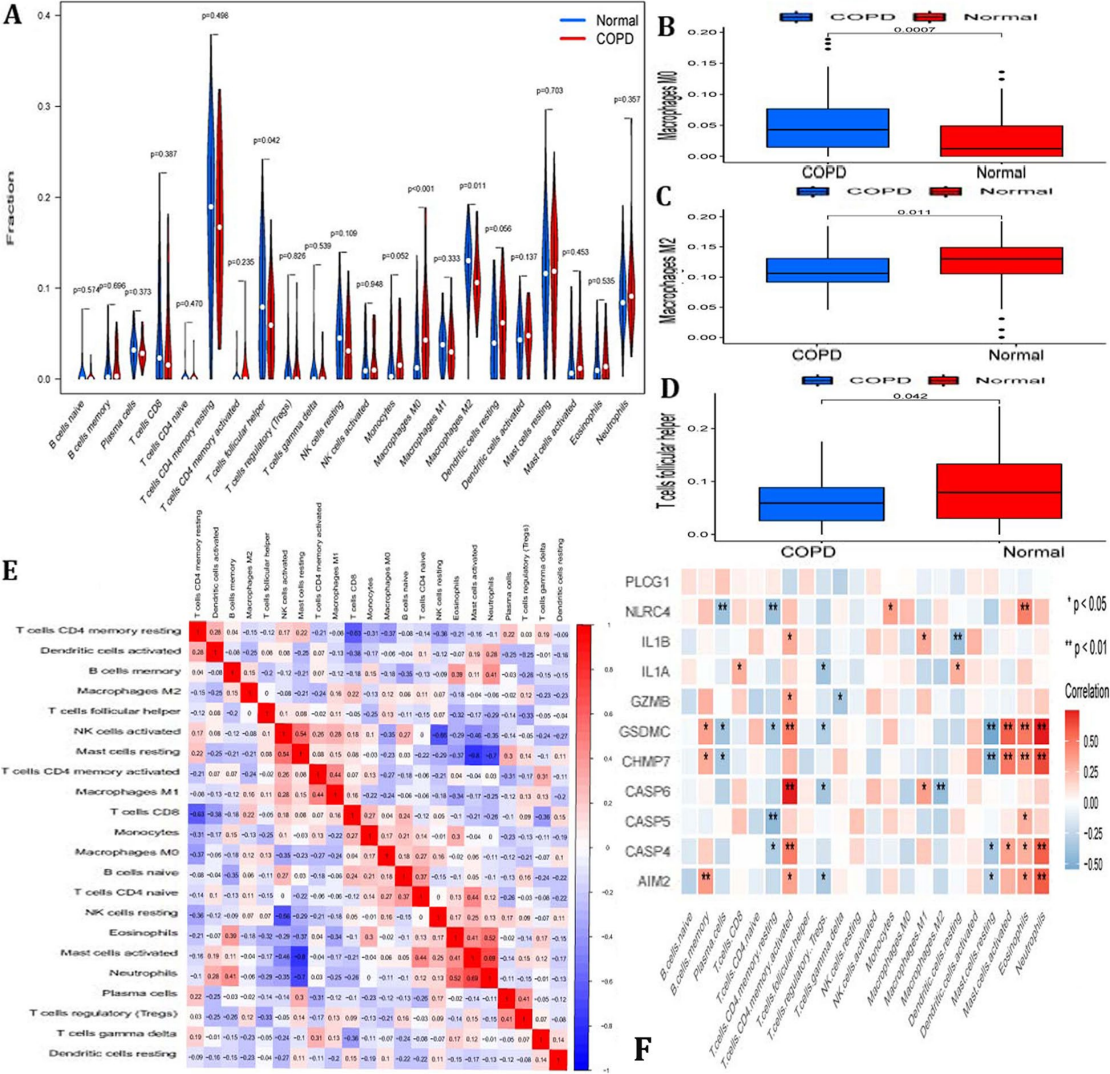
Immune Landscape of COPD **A** The fraction of 22 types of immune cells in COPD patients and normal cases. **B**-**D** The distribution difraction of macrophages M0, macrophages M2 and T cells follicular helper in COPD patients and normal cases. **E** The correlation of 22 types of immune cells in COPD samples. **F** the correlation of 9 COPD associated pyroptosis-related gene expression and the abundance of immune cell infiltration. *P*-values are shown as: *p < 0.05; **p < 0.01

The samples of test set were performed for the analysis of CIBERSORT. In Fig. 7A, the proportion of 22 types of immune cells in COPD patients and normal cases was shown in a violin diagram by Wilcoxon test. The distribution proportion of macrophages M0 (*p* = 0.0007), macrophages M2 (*p* = 0.09) and T cells follicular helper (*p* = 0.042) have significant variation in COPD patients and normal cases (Figue 7B-D). Then, we evaluated the correlation between 22 types of immune cells in COPD samples (Fig. 7E). We also explored the correlation of the expression of 9 COPD-associated pyroptosis-related genes and the abundance of immune cell infiltration (Fig. 7F). As the result, the expression of GMZB was negatively associated with the abundance of Macrophages.M2. Eosinophils had a positively relation with the expression of IL1B, CHMP7, CASP5, CASP4. The result suggested that the expression of 9 COPD-associated pyroptosis-related genes may influence the infiltration of these immune cells.

## Discussion

In light of data suggesting that about 20% of COPD patients have experienced exacerbations per year, which resulted in significant treatment costs [30, 31]. In routine clinical work, different treatment modalities will be chosen according to disease severity from COPD [32]. However, the level of standardized treatment, pulmonary rehabilitation, and pneumococcal vaccination remain low, strengthening the work of the diagnostic and management levels is urgent [33, 34].

Pyroptosis, a form of programmed cell death and an innate immune mechanism, triggers by the activation of the ASC/caspase-1 pathway [35]. Similar to apoptosis, both would appear nuclear condensation, DNA fragmentation and TUNEL staining [36]. However, apoptosis maintains membrane integrity, while pyroptosis would lead to pore formation and membrane rupture resulting in the release of intracellular contents and inflammatory factors [37]. Based on the current study, overactivated inflammasome in the process of pyroptosis would accelerate the progression of COPD [3].

In our research, 9 candidate genes were identified to be associated with the occurrence or development of COPD. According to the current study, the increases in reactive oxygen species (ROS) and caspase-4 have been reported to involved in induced lung cell death. Caspase-4 also highly expresses in the blood of both smokers and COPD patients up to lung cancer patients [38]. As for Caspase-5, it might be a suppressor gene of highly metastatic potential in lung cancer. GZMB levels as well as T cells expressing GZMB are increased in bronchoalveolar lavage (BAL) fluid of COPD patients. When it comes to the functions of IL-1 in lung disease, a study showed that IL-1 has a complex modulatory role in the development of pulmonary fibrosis, acting on structural cells by dampening collagen production and furthermore balancing the pro-fibrotic and pro-inflammatory actions of the immune system [39]. It has also been reported that NLRC4 could produce IL-1R antagonist (IL-1Ra) via NF-κB, to bind IL-1β, delaying the progression of fibrogenesis [40]. And NLRC4 signaling contributes to several bacteria-induced lung inflammation, even tuberculosis [41, 42]. CASP6 were also found to be increased in the progression of fibrogenesis, but the detailed mechanism need be further explored [43]. In smoke-induced experimental COPD, AIM2 plays a vital role in regulating lung neutrophilic inflammation and caspase-1 activation in these cells following recruitment [44]. Additionally, the activation of AIM2 inflammasome correlates with the disease severity and the protein redistribution between the nucleus and cytoplasm [45]. Research effort is currently dedicated to explore the relationship between pyroptosis-related genes and lung cancer, which concluded that overexpression of GSDMC has a predictive ability in the prognosis of lung adenocarcinoma [46]. Research effort is currently dedicated to explore the relationship between pyroptosis-related genes and lung cancer, which concluded that overexpression of GSDMC has a predictive ability in the prognosis of lung adenocarcinoma [47],and PLCG1 as a critical mediator of the FGFR1 signaling, plays a vital in regulating neuroendocrine differentiation in small cell lung cancer [48].

In our research, twenty-six COPD key genes and 9 COPD-associated pyroptosis-related genes were identified. The PPI analysis and gene correlation analysis were applied to depict their relationship clearly. KEGG and GO analysis of COPD key genes and COPD-associated pyroptosis-related genes respectively have revealed the main mechanisms as well as pyroptosis-related mechanisms in COPD. The expression of 9 COPD-associated pyroptosis-related genes in different grade suggested that pyroptosis could contribute to the progression of COPD. The immune environment of COPD was also explored. Moreover, the relationship of pyroptosis-related genes and the expression of immune cell were also be shown in the end, which reveal that pyroptosis could influence immune microenvironment of COPD. Because pyroptosis is linked tightly to development of COPD, therefore, understanding of pyroptosis-related genes potential in treatment generated high expectations. This study may provide new insight into the novel therapeutic targets for COPD clinical treatment.

Although this particular relationship was explored to several extent, some shortcomings and limitations in our study should be recognized. First, this study used retrospective data, which may have some heterogeneity among patients. Therefore, more prospective cohort studies in larger populations are needed to test the result. Second, external validation via other clinical datasets hasn’t been implemented and the biological mechanism of some pyroptosis-related genes has not been fully elucidated. Thus, more external experiments will be conducted in further research work.

Token together, our study concluded that pyroptosis may contribute to the occurrence and development of COPD.

## Conclusion

In our research, we have explored the relationship between pyroptosis-related genes and COPD key genes and the significant differential expression of COPD-associated pyroptosis-related genes in different grades to confirm that pyroptosis could contribute to the progression of COPD. The immune environment of COPD, the relationship of pyroptosis-related genes and the expression of immune cell were also be shown in the end, which reveal that pyroptosis could influence immune microenvironment of COPD. Because pyroptosis is linked tightly to the development of COPD, therefore, understanding of pyroptosis-related genes potential in treatment generated high expectations. This study may provide new insight into the novel therapeutic targets for COPD clinical treatment.

## Supplementary Information

**Additional file 1: Supplemental Table 1.** Forty-seven pyroptosis-related.

**Additional file 2: Supplemental Table 2.** The clinical information of Train set samples and Test set sample.

**Additional file 3: Supplemental Table 3.** Nine COPD-associated pyroptosis-related genes selected by differential expression analysis.

**Additional file 4: Supplemental Table 4.**

**Additional file 5: Supplemental Table 5.**

## Data Availability

The Data used in the current study was obtained from publicly available repository. The datasets generated or analyzed during the current study are available in the GEO database (GSE5058, GSE8545, GSE11906, GSE20257 and GSE69818) repository, https://www.ncbi.nlm.nih.gov/geo/.
